# High-throughput polymorphism detection and genotyping in *Brassica napus* using next-generation RAD sequencing

**DOI:** 10.1186/1471-2164-13-281

**Published:** 2012-06-24

**Authors:** Anja Bus, Jochen Hecht, Bruno Huettel, Richard Reinhardt, Benjamin Stich

**Affiliations:** 1 Max Planck Institute for Plant Breeding Research, Carl-von-Linné-Weg 10, 50829 Cologne, Germany; 2 Berlin-Brandenburg Center for Regenerative Therapies, Charité-Universitaetsmedizin Berlin, Augustenburger Platz 1, 13353 Berlin, Germany; 3 Max Planck Genome Centre Cologne, Carl-von-Linné-Weg 10, 50829 Cologne, Germany

**Keywords:** *Brassica napus*, Restriction-site associated DNA, Next-generation sequencing, Single nucleotide polymorphism, Genotyping by sequencing, Genetic diversity

## Abstract

**Background:**

The complex genome of rapeseed (*Brassica napus*) is not well understood despite the economic importance of the species. Good knowledge of sequence variation is needed for genetics approaches and breeding purposes. We used a diversity set of *B. napus* representing eight different germplasm types to sequence genome-wide distributed restriction-site associated DNA (RAD) fragments for polymorphism detection and genotyping.

**Results:**

More than 113,000 RAD clusters with more than 20,000 single nucleotide polymorphisms (SNPs) and 125 insertions/deletions were detected and characterized. About one third of the RAD clusters and polymorphisms mapped to the *Brassica rapa* reference sequence. An even distribution of RAD clusters and polymorphisms was observed across the *B. rapa* chromosomes, which suggests that there might be an equal distribution over the *Brassica oleracea* chromosomes, too. The representation of Gene Ontology (GO) terms for unigenes with RAD clusters and polymorphisms revealed no signature of selection with respect to the distribution of polymorphisms within genes belonging to a specific GO category.

**Conclusions:**

Considering the decreasing costs for next-generation sequencing, the results of our study suggest that RAD sequencing is not only a simple and cost-effective method for high-density polymorphism detection but also an alternative to SNP genotyping from transcriptome sequencing or SNP arrays, even for species with complex genomes such as *B. napus*.

## Background

Rapeseed (*Brassica napus*) is an important oilseed and energy crop. Its allotetraploid genome fully comprises the two genomes of the species *Brassica rapa* (A genome) and *Brassica oleracea* (C genome), and the estimated genome size of *B. napus* is about 1.2 Gbp [1]. The large and complex nature of the *B. napus* genome makes the species a challenging target for genomic research.

The knowledge of sequence variation and especially the availability of a high number of molecular markers for a species serve as tools for various plant genetics approaches, such as marker-assisted backcrossing or map based cloning [2]. But also studies of linkage disequilibrium (LD) in *B. napus* have indicated that ten thousands of molecular markers might be necessary for comprehensive whole-genome association studies (GWAS) in this species [3,4]. Owing to the size and complexity of the *B. napus* genome as well as the lack of a public reference genome sequence, whole-genome resequencing is extremely elaborate and costly, and therefore currently not the method of choice for assessing sequence variation and identifying DNA polymorphisms in *B. napus*. One possibility to reduce the amount of the target sequence is transcriptome sequencing. In oilseed rape, Trick *et al.*[5] identified more than 40,000 putative single nucleotide polymorphisms (SNPs) between the cultivars *`*Tapidor’ and *`*Ningyou 7’ through transcriptome sequencing using the Illumina Solexa platform. However, the transcriptome merely provides information from coding regions, where nucleotide diversity is lower compared to non-coding regions [6,7].

An alternative approach to reduce the complexity of a genome is the use of restriction enzymes. This principle is the basis for the restriction-site associated DNA (RAD) sequencing technology, which allows parallel sequencing of millions of DNA fragments flanking individual restriction enzyme sites. The RAD approach has been successfully applied in a number of organisms, including crop species like barley [8], perennial ryegrass [9], maize [10], eggplant [11], and artichoke [12]. Nelson *et al.*[13] sequenced a library of eight diverse sorghum accessions to detect SNPs and study their genomic distribution, and they found 283,000 SNPs based on an alignment to the reference genome. To our knowledge, the RAD method has not been applied in earlier studies to identify sequence polymorphisms in an allotetraploid species like *B. napus*.

The DNA polymorphisms identified by RAD sequencing need to be characterized to facilitate subsequent applications. Due to the availability of the *B. rapa* reference sequence and the annotated 95K Brassica unigene (UG) dataset (http://brassica.bbsrc.ac.uk/) it is possible to carry out BLAST searches against these datasets, examine the functions of those genes with DNA polymorphisms for which BLAST hits were obtained, and also check the genome-wide distribution of DNA polymorphisms across the *B. rapa* chromosomes. Furthermore, the categorization of genes with DNA polymorphisms by Gene Ontologies (GO) helps to understand signatures of selection.

Here, we describe the construction of a comprehensive, well-characterized set of DNA polymorphisms in *B. napus* based on RAD sequencing. The objectives of our study were to (i) establish genomic RAD tags from eight diverse *B. napus* inbred lines for high-density polymorphism detection and genotyping, (ii) validate a subset of the identified polymorphisms by Sanger sequencing, and (iii) characterize the RAD tags according to BLAST searches against genomic databases of *B. rapa* and *B. napus*.

## Results

**Figure 1:**
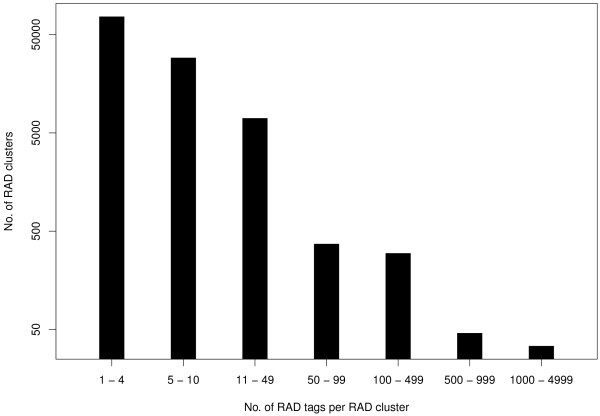
**Restriction-site associated DNA (RAD) clusters with different numbers of RAD tags across eight *B. napus* inbreds.**

**Figure 2:**
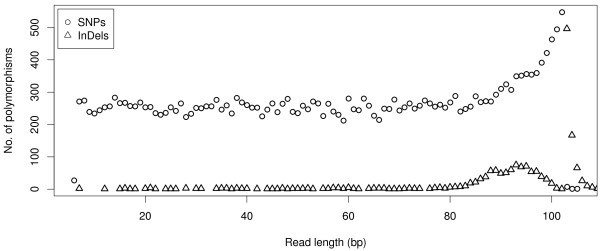
Distribution of restriction-site associated DNA polymorphisms (single nucleotide polymorphisms (SNPs) and insertions/deletions (InDels)) along the read length.

**Figure 3:**
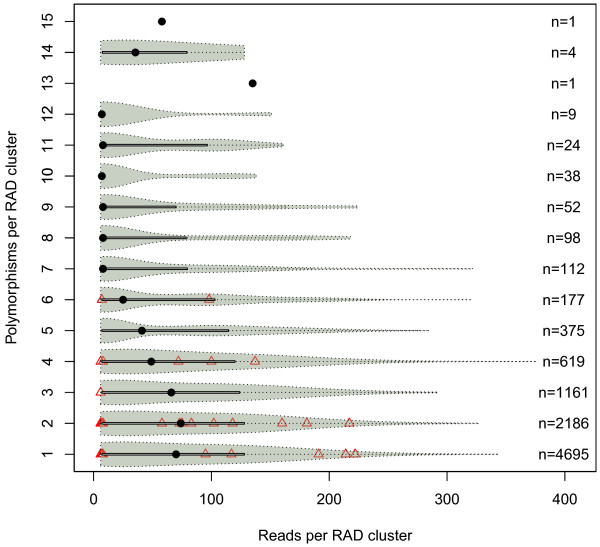
**Number of polymorphisms and number of reads per restriction-site associated DNA (RAD) clusters across eight *B. napus* inbreds.** Violin plot denotes 9,552 RAD clusters with single nucleotide polymorphisms (SNPs), and triangles denote 59 RAD clusters with insertions/deletions (InDels). Black dots denote the median number of reads per RAD cluster.

**Figure 4:**
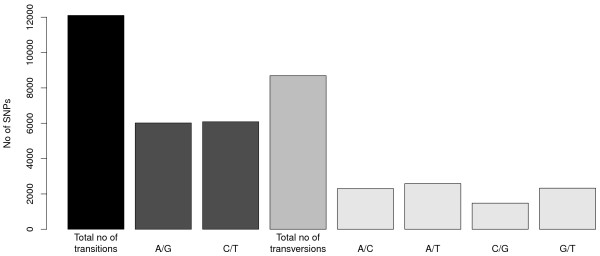
**Transitions and transversions within 20,795 biallelic single nucleotide polymorphisms (SNPs) detected among eight *B. napus* inbreds.**.

Sequencing the *Kpn*I library generated more than 26 million single-end reads of 120 bp length (Table 1). Sequence data have been deposited in the NCBI Sequence Read Archive under submission number SRA052686. More than 99% of the reads contained the barcode, and more than 95% contained the restriction overhang, where one mismatch was allowed (Table 1). After clustering reads to RAD tags and removing RAD tags with more than 200 reads, the number of RAD tags per inbred ranged from 100,420 to 127,681. Altogether 636,179 RAD tags were assigned to 113,221 RAD clusters. RAD clusters with one to four RAD tags occurred most frequently, and there was a gradual decrease in the number of RAD clusters with larger numbers of RAD tags (Figure 1). After the filtering steps to remove intergenomic polymorphisms, nearly 33,000 polymorphisms were detected (Table 1). SNPs and insertions/deletions (InDels) identified at positions 7 to 88, and 7 to 80 at a read, respectively, occurred with equal frequency at each position (Figure 2), and only those were used for all further analyses. Polymorphisms outside the aforementioned read positions were more numerous due to a higher number of sequencing errors and were therefore discarded. Afterwards, altogether 20,835 SNPs were detected from 9,552 RAD clusters, and 125 InDels were detected from 59 RAD clusters (Table 1, Additional file 1). A decrease in the number of reads per RAD cluster also resulted in a decreasing number of polymorphisms per RAD cluster (Figure 3). Of all SNPs, 40 were triallelic. The remaining 20,795 SNPs were biallelic and consisted of 58.2% transitions and 41.8% transversions (Figure 4), providing a ratio of 1.39. The number of different transitions was balanced (6,018 A/G and 6,084 C/T), and the number of transversions ranged from 1,480 (C/G) to 2,583 (A/T).

**Figure 5:**
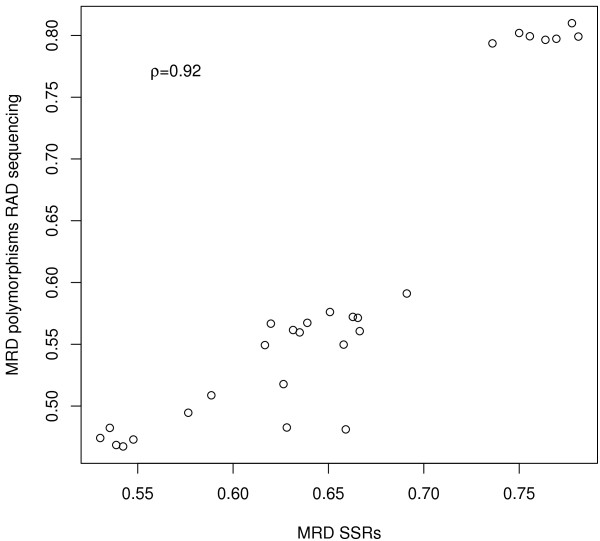
**Correlation of Modified Roger’s distances (MRD) between pairs of eight *B. napus* inbreds.** MRD estimates were determined with simple sequence repeat (SSR) markers and polymorphisms from restriction-site associated DNA (RAD) sequencing.

The correlation between pairwise modified Roger’s distance (MRD) estimates among all inbreds based on simple sequence repeat (SSR) data from a previous study [3] and based on DNA polymorphism data from RAD sequencing was *ρ* = 0.92 (Figure 5). The 16 RAD clusters selected for validation comprised 31 SNPs, of which 26 (83.9%) were verified to be polymorphic. Out of the five non-polymorphic SNPs, four belonged to one specific RAD cluster. RAD and Sanger sequencing information did not agree for 13.1% of the inbred-allele combinations observed for the 31 SNPs.

**Table 1:** Restriction-site associated DNA (RAD) sequencing statistics summary of a *Kpn*I library from eight *B. napus* inbred lines

**Statistic**	**Number**
Total Illumina reads, x 1^06^	26.47
Total reads containing barcode	
x 1^06^ (1 mismatch allowed)	26.33
Total reads containing restriction	
overhang x 1^06^ (1 mismatch allowed)	25.16
Reads per inbred containing restriction	
overhang x 1^06^ (1 mismatch allowed) (range)	2.08-4.14
RAD tags per inbred with <200	
reads (range)	100,420-127,681
Mean of reads per RAD tag with ≤200 reads (range)	13.74-22.43
Median of reads per RAD tag with ≤200	
reads (range)	10-17
Total RAD clusters	113,221
Total polymorphisms	32,839
Single nucleotide polymorphisms (SNPs)	
from positions 7-88 bp	20,835
RAD clusters from which SNPs were derived	9,552
Insertions/Deletions (InDels) from positions 7-80 bp	125
RAD clusters from which InDels were derived	59
Reads per SNP-inbred combination (median)	9.7
Reads per InDel-inbred combination (median)	1.0

**Figure 6:**
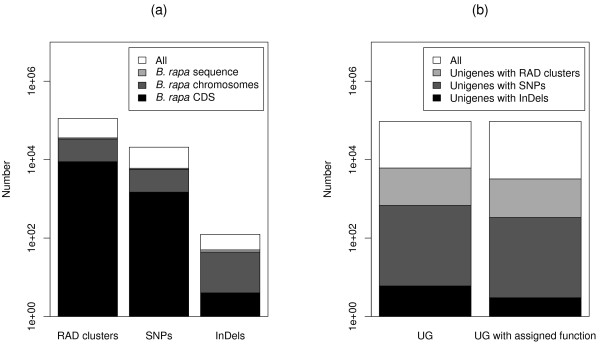
**Representation of *B. napus* restriction-site associated DNA (RAD) information and unigenes (UG).** (**a**) RAD clusters, single-nucleotide polymorphisms (SNPs), and insertions/deletions (InDels) in the *B. rapa* sequence, *B. rapa* chromosomes, and *B. rapa* coding sequence (CDS) after BLAST searches and (**b**) UG with RAD clusters and polymorphisms, and UG with RAD clusters and polymorphisms that were assigned a function after BLAST searches against the UniProtKB/Swiss-Prot dataset.

**Figure 7:**
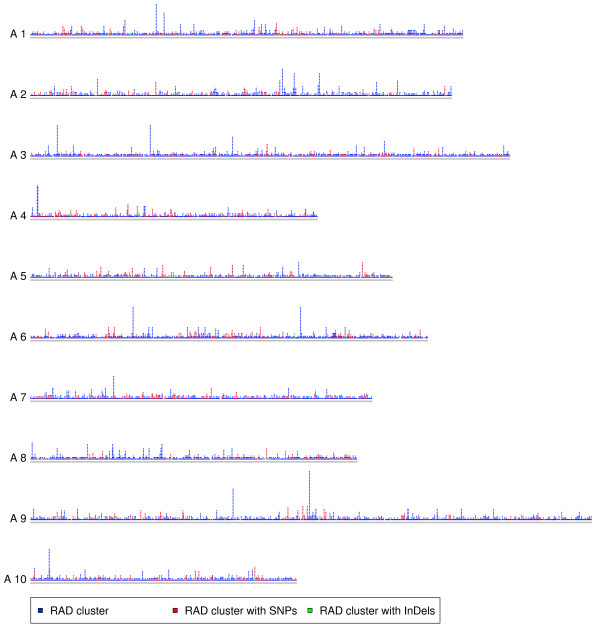
**Distribution of restriction-site associated DNA (RAD) information in *B. rapa* .** Dispersal of RAD clusters, RAD clusters with single nucleotide polymorphisms (SNPs), and RAD clusters with insertions/deletions (InDels) across the ten chromosomes of *B. rapa*.

Of all RAD clusters and polymorphisms, 35,960 RAD clusters (31.8%), 6,042 SNPs (29.0%), and 50 InDels (40%) were found in the *B. rapa* sequence data (Figure 6(a)), 33,749 RAD clusters (29.8%), 5,687 SNPs (27.3%), and 44 InDels (35.2%) were found in the *B. rapa* chromosome data, and 8,873 RAD clusters (7.8%), 1,482 SNPs (7.1%), and 4 InDels (3.2%) were found in the *B. rapa* coding sequence (CDS) data after BLAST searches. The transition/transversion ratio was 1.45 calculated for the *B. rapa* sequence data and 1.60 for the *B. rapa* CDS data. RAD clusters and polymorphisms were distributed evenly across the ten *B. rapa* chromosomes (Figure 7).

**Figure 8:**
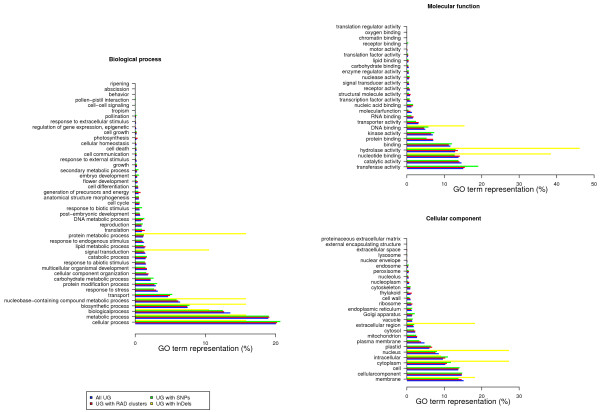
**Gene Ontology (GO) term representation.** GO term representation (%) of all *B. napus* unigenes (UG), UG with restriction-site associated DNA (RAD) clusters, UG with single nucleotide polymorphisms (SNPs), and UG with insertions/deletions (InDels) according to GO slim categories.

Altogether 9,469 RAD clusters (8.4%), 1,245 SNPs (6.0%), and 10 InDels (8.0%) were found at least once in the Brassica UG dataset after BLAST searches (Table 2). In the search against all 94,558 Brassica UG, we found for 6,140 UG RAD clusters, for 678 UG SNPs, and for 6 UG InDels (Figure 6(b)). A total of 3,231 UG with RAD clusters (52.6%), 335 UG with SNPs (49.4%), and 3 UG with InDels (50.0%) could be assigned a function after BLAST searches against the UniProtKB/Swiss-Prot dataset. The GO term representation was balanced between all UG, UG with RAD clusters, and UG with SNPs, whereas GO terms for UG with InDels were generally either over- or underrepresented (Figure 8).

**Table 2:** Number of occurrence of restriction-site associated DNA (RAD) clusters, single nucleotide polymorphisms (SNPs), and insertions/deletions (InDels) in the Brassica unigene dataset

	**Frequency of occurrence**							
	**0**	**1**	**2**	**3**	**4**	**5**	**6**	**7**
RAD clusters	103,752	8,314	1,019	100	25	8	1	2
SNPs	19,590	1,132	109	3	1	0	0	0
InDels	115	8	2	0	0	0	0	0

## Discussion

### RAD sequencing and representation of the *B. napus* genome

In our study, 113,221 RAD clusters were sequenced (Table 1). With a length of 102 bp per RAD cluster after trimming and the fact that only a very limited number of pairs of restriction sites of *Kpn*I occur within 102 bp, our results indicate that we obtained sequence information from eight genotypes which represents about 1% of the *B. napus* genome. So far, comparative sequencing of such a large part of the *B. napus* genome has not been performed in more than two genotypes.

To increase the genome representation through RAD sequencing approaches in *B. napus* further, it might be useful to apply paired-end sequencing methods. Also, sequencing libraries constructed with restriction enzymes cutting more frequently than *Kpn*I would result in a higher genome representation. Alternatively, more than one library prepared with different restriction enzymes could be sequenced on separate lanes of a flow cell to improve the read yield. The latter two approaches have been demonstrated to be useful in sorghum [13], and explain why a substantially larger percentage of the genome could be targeted by RAD sequencing in that study. However, when using more than one enzyme and sequencing a number of different libraries, the necessity for enzyme-specific adapters will increase the total costs. This problem can be circumvented by using double digest RAD sequencing [14], where DNA is digested with different restriction enzymes simultaneously.

### Genome-wide distribution of RAD polymorphisms

The RAD clusters detected in our study for *B. napus* were tested for their presence in the known *B. rapa* genome sequence. We found only about one third of the RAD clusters in the *B. rapa* sequence, and slightly less in the *B. rapa* chromosome data (Figure 6(a)). The *B. rapa* genome has been reported to have a size of about 529 Mbp [15,16], whereas the size of the *B. oleracea* genome has been described to be larger, namely 599-696 Mbp [1,15]. Hence, it is expected that the number of RAD clusters mapping to the *B. oleracea* genome is higher compared to the number of RAD clusters mapping to the *B. rapa* genome, given that the G/C content, which influences the number of restriction fragments and consequently also the number of RAD clusters, is identical for the two species. Results from earlier studies show that this is the case, as they found that the G/C content was 35.4% in *B. rapa*[17], and 36.0% in *B. oleracea*[18]. Despite the aforementioned genome size of *B. rapa*, the published *B. rapa* reference sequence [19] has a size of 284 Mbp. We therefore conclude that a considerable part of the *B. rapa* genome sequence is not part of the reference sequence, which, together with the smaller genome size when compared to *B. oleracea*, explains the fraction of RAD clusters we found in the *B. rapa* sequence. In addition, the BLAST searches might have been affected by matches against low complexity regions.

We observed an equal distribution of RAD clusters across the *B. rapa* chromosomes (Figure 7). Our finding suggests that we can expect a similar distribution of RAD clusters with regard to the chromosomes of the unknown C genome. This result in turn suggests that polymorphisms detected from RAD clusters are also uniformly distributed across the *B. napus* genome, which makes them an important resource not only for GWAS but also other applications like high-resolution linkage mapping.

### Polymorphism detection and genotyping

In the examined eight *B. napus* inbreds, we observed for 113,221 RAD clusters a total of 20,835 SNPs and 125 InDels (Table 1). Considering that for SNPs, 82 bp (positions 7-88) and for InDels, 74 bp (positions 7-80) of each RAD cluster were regarded, we detected SNPs from a total of 9,284,122 bp and InDels from a total of 8,378,354 bp. Hence, we found one SNP every 446 bp and one InDel every 67,027 bp for a very diverse set of *B. napus*. The study by Westermeier *et al.*[6] on six *B. napus* winter oilseed rape varieties observed with one SNP every 247 bp a slightly higher polymorphism frequency. This might be due to sampling effects because candidate sequences with a total length of 21.4 kb of the *B. napus* genome were investigated. However, with one InDel every 3,583 bp, the study by Westermeier *et al.*[6] observed a drastically higher InDel frequency. This is because the relatively short reads of 102 bp in our study in combination with the unavailability of a reference sequence are not powerful for the detection of InDels.

Trick *et al.*[5] estimated the overall sequence polymorphism rate between the transcriptomes of the two cultivars ‘Tapidor’ and ‘Ningyou 7’ to be one SNP per 2,130 bp based on a minimum read depth of eight, or 1,195 bp based on a minimum read depth of four. The reason for the lower SNP frequency in that study when compared to ours is most likely the derivation of SNPs from the less polymorphic coding region. Moreover, only two inbreds were investigated in the study by Trick *et al.*[5], which leads to an underestimation of the SNP frequency in a species.

### Characterization of polymorphisms

The overall representation of different transitions (58.2% (total), 49.7% (A/G), 50.3% (C/T)) and transversions (41.8% (total), 26.5% (A/C), 29.7% (A/T), 17.0% (C/G), and 26.8% (G/T) (Figure 4)) we observed was in good accordance with those detected by Barchi *et al.*[11] in eggplant through RAD sequencing (transitions: 49.7% (A/G), 50.3% (C/T), transversions: 24.0% (A/C), 28.5% (A/T), 19.9% (C/G), 27.6% (G/T)). Both studies showed a preponderance of transitions over transversions, which has been observed earlier in various species [20,21]. However, the ratio of total transitions/transversions was with 1.39 lower in our work compared to 1.65 in the study by Barchi *et al.*[11]. On the other hand, in a study on DNA polymorphism in *B. rapa*[22], more than 21,000 SNPs were discovered and characterized in eight diverse genotypes, and a transition/transversion ratio of 1.03 was observed. We therefore conclude that the ratio we found fits into the range of observations from comparable studies. The aforementioned research [22] furthermore found a transition/transversion ratio of 1.03 in exons and introns versus a ratio of 1.63 in exons only. Hence, our observation of a lower ratio in the *B. rapa* sequence data (1.45) compared to that in the coding sequence data (1.60) is in line with earlier findings from the species *B. rapa*.

The BLAST search against the UG set allowed us to identify the fractions of unigenes with RAD clusters and polymorphisms. For this search it was useful to apply a large sample size, therefore we refrained from a differentiation between the *B. napus* A and C genomes for this part of the study. Consequently, also the BLASTX search for each UG against the UniProtKB/Swiss-Prot dataset was based on the *B. napus* data. We observed a tight correlation between the GO term representations of all UG and UG with RAD clusters and polymorphisms, except for an apparent over- and underrepresentation of GO terms for UG with InDels (Figure 8). This observation is due to the small number of that polymorphism type in this work. Hence, the results of our study revealed no signature of selection with respect to the distribution of polymorphisms within genes belonging to a specific GO category.

### Verification of polymorphisms

In our study, 26 out of 31 SNPs (84%) were verified to be polymorphic according to the Sanger sequencing information. However, four out of the five non-polymorphic SNPs in our work were from one specific RAD cluster. If this RAD cluster was disregarded, the percentage of verified polymorphisms would be close to 100%. It is not obvious why the SNPs from this RAD cluster could not be validated. The RAD cluster was based on a low number of reads (data not shown), but so were other RAD clusters from which SNPs could be validated. A possible reason might be that this RAD cluster comes from a region which is similar between the *B. rapa* and *B. oleracea* genomes. The Illumina sequencing approach does not allow the assignment of the reads to the two different genomes and, thus, hemi-SNPs were considered as SNPs. In contrast, with Sanger sequencing, only the SNPs in one of the genomes have been targeted. However, this requires further research.

The high correlation (*ρ*=0.92) of MRD determined with SSR markers and RAD polymorphisms between pairs of the eight inbreds examined in our study (Figure 5) indicated that the RAD polymorphisms identified are likely to be true polymorphisms. Furthermore, this observation is strongly supported by the verification data of 31 SNPs by Sanger sequencing, where only 13.1% of the inbred-allele combinations observed for the 31 SNPs did not agree. Trick *et al.*[5] also validated candidate SNPs in their study. Out of nine SNPs that had been PCR amplified previously, eight had been called as hemi-SNPs according to the transcriptome sequencing data. Four of the hemi-SNPs (44.4%) were confirmed, four of the hemi-SNPs (44.4%) were uninformative, and the ninth putative SNP (11.1%) was contradicted. Moreover, Trick *et al.*[5] found eight out of nine SNPs (88.9%) in the aligned regions to be polymorphic in both data sets. Therefore, the number of validated polymorphic RAD clusters and inbred-allele combinations in our study provides a good rate of correctly called polymorphisms.

## Conclusions

We applied the RAD sequencing strategy in *B. napus*, which allowed us to detect more than 20,000 SNPs and simultaneously genotype eight inbred lines. Having sequenced about 1% of the *B. napus* genome, we managed to carry out comparative sequencing of a large part of the genome in a diverse set of the species. The equal dispersal of identified polymorphisms across the genome underlines the suitability of RAD sequencing for obtaining markers useful for GWAS and linkage mapping purposes. RAD sequencing reduces the complexity of the genome and detects sequence variation from coding and non-coding regions across the genome. Finally, the successful verification of SNP information demonstrates that our RAD sequencing experiment detected true polymorphisms with a high rate of correctly called alleles.

## Methods

### Plant material and DNA isolation

The following eight *B. napus* inbred lines, which represent eight germplasm types as described by [3], were used for our study: *`*Express 617’ (German winter oilseed rape), *`*PSA 12’ (newly resynthesized Canadian spring oilseed rape), *`*Devon Champion’ (British swede cultivar), *`*Grüner Schnittkohl’ (German vegetable type), *`*Canard’ (British winter fodder rape), *`*Tira’ (German spring fodder rape), *`*SWU Chinese 9’ (Chinese semi-winter oilseed rape), and *`*PI 271452’ (unspecified line from India). DNA was extracted from leaf tissue using the DNeasy Plant Mini Kit (Qiagen).

### RAD library preparation and sequencing

The RAD library of the eight inbreds was prepared for single-end sequencing according to Baird *et al.*[23] with the following modifications: Barcodes were 5 bp long, being at least two mutational steps separated from each other with regard to the first four bases, followed by the fifth checksum base. A total of 2 µg genomic DNA of each inbred was digested for 30 min at 37°C in a 50 µl reaction with 20 units of *Kpn*I (New England Biolabs). Samples were inactivated by purification with Qiaquick spin columns (Qiagen). The RAD library was sequenced on one lane of the Illumina GAIIx system with 120 bp reads at the Charité, Berlin, following the manufacturer’s protocol.

### Sequence analysis and polymorphism detection

Reads were separated by barcode if applicable and trimmed by 17 bases at the 3’ end. Reads with more than ten bases showing a quality score below 30 were discarded. Due to the unavailability of a *B. napus* reference sequence, we applied the following de novo RAD analysis procedure. The software RADtags from the software package RADtools [24] was used to cluster the reads of each of the eight inbreds into RAD tags. The options for this clustering were (i) bases with quality scores below 20 were ignored, (ii) the number of mismatches allowed for a RAD tag to be added to a RAD cluster was three, and (iii) RAD tags with less than three or more than 200 reads were discarded. Afterwards, RAD tags were merged across all inbreds into RAD clusters if the maximum number of mismatches was twelve. For this step, the software tool RADmarkers was used.

The RAD tags present at RAD clusters were screened for SNPs and InDels. The principle was that polymorphisms between inbreds were considered, whereas polymorphisms within inbreds were only considered if one of the alleles was specific to one inbred. In detail, the following filters, which take the allotetraploid nature of the *B. napus* genome into account, were applied: (i) polymorphisms in RAD clusters were discarded if at least one inbred carried more than two RAD tags, because this is higher than what would be expected based on the allotetraploid genome of *B. napus*, (ii) the sequence information of inbreds was discarded for a RAD cluster if more than one inbred carried two RAD tags, because in this case no assignment of SNPs to one homoeologue is possible, and (iii) if in one RAD cluster one inbred carried two RAD tags, the polymorphisms were only considered if the alleles were specific to one inbred (cf. [5]). Furthermore, all polymorphisms were discarded from further analyses if the corresponding allele information was missing for more than two inbreds. Pairwise MRD estimates between all eight inbreds were calculated according to Wright [25].

### Sequence and polymorphism characterization

A BLASTN search was performed for the consensus sequences of all RAD clusters as well as only for those that contain SNPs or InDels against two data sources using an expectation cutoff value of 1e-15. Further settings were chosen as follows: cost to open a gap = 25, cost to extend a gap = 10, reward for a nucleotide match = 2, penalty for a nucleotide mismatch = -3, word size = 56, megablast search = T, number of alignments to show = 1. The first search was against the entire *B. rapa* genome sequence (which includes sequence data that were not assigned to the ten pseudochromosomes), the chromosome data, and the CDS data of the genotype Chiifu-401 [26]. Similarly, a search against the 95K Brassica species UG dataset (http://brassica.bbsrc.ac.uk/) was performed. A BLASTX search was carried out for each UG against the UniProtKB/Swiss-Prot dataset, adopting an E-value of 1e-10 in order to assign the UGs a function. The GO terms of different subsets of UG were mapped to higher level categories (plant GO slim) using GOSlimViewer [27] according to the three principal GO categories. If not stated otherwise, all analyses were performed using the software R [28].

### Polymorphism verification

Sixteen polymorphic RAD clusters that were found to be located in *B. napus* UGs were randomly selected. Primers were designed to flank the entire RAD cluster, yielding products of 160-391 bp. The target sequence was amplified in all eight genotypes. PCR conditions were as follows: 94°C for 3 min; 35 cycles of 94°C for 1 min, 59°C for 1 min, 72°C for 1 min; and 72°C for 5 min, afterwards samples were held at 4°C. Sanger sequencing of PCR products was carried out by the Max Planck Genome Centre Cologne following standard protocols. The proportion of DNA polymorphisms from RAD sequencing that were found back in the Sanger sequencing results as well as the proportion of identical allele calls across all inbreds and DNA polymorphisms were calculated.

## Abbreviations

LD = Linkage disequilibrium; GWAS = Whole-genome association study; SNP = Single nucleotide polymorphism; RAD = Restriction-site associated DNA; UG = Unigene; GO = Gene Ontology; InDel = Insertion/deletion; MRD = Modified Roger’s distance; SSR = Simple sequence repeat; CDR = Coding sequence.

## Competing interests

The authors declare that they have no competing interests.

## Authors’ contributions

BS designed and supervised the project. AB analyzed the data. JH, BH, and RR supported the RAD library construction and coordinated the Illumina sequencing procedure. AB and BS wrote the manuscript. All authors read and approved the final manuscript.

## Supplementary Material

Additional file 1 **Restriction-site associated DNA (RAD) cluster sequence information flanking 20,835 single nucleotide polymorphisms (SNPs) and 125 insertions/deletions (InDels) identified through RAD sequencing.** All polymorphisms are indicated in brackets. Click here for file
